# An Ultra-Compact MEMS Pirani Sensor for In-Situ Pressure Distribution Monitoring

**DOI:** 10.3390/mi13101686

**Published:** 2022-10-07

**Authors:** Lan Zhang, Jian Lu, Hideki Takagi, Sohei Matsumoto, Eiji Higurashi

**Affiliations:** Device Technology Research Institute, National Institute of Advanced Industrial Science and Technology (AIST), Tsukuba 305-8564, Japan

**Keywords:** MEMS, Pirani sensor, vacuum test, simple process, lift-off

## Abstract

In this study, we designed a microelectromechanical system (MEMS) Pirani vacuum sensor with a compact size. Specifically, the sensor was successfully fabricated based on the Pirani principle and using a commercial eight-inch MEMS foundry process. The sensor fabrication process was carried out using only four photomasks and the proposed sensor had an ultra-compact fabricated size (<2.2 × 2.2 mm^2^). A vacuum measurement system was set up to comprehensively evaluate the fabricated sensors. The results demonstrated that the MEMS Pirani vacuum sensor has a high responsivity in the low-pressure domain from 100 Pa. The proposed sensor with a 953.0-Ω heater exhibited an average responsivity of 11.9 mV/Pa in the preferred range of 100 to 7 Pa and 96.0 mV/Pa in the range of 7 to 1 Pa. The sensor may be potentially suitable in many applications, such as vacuum indicators for processing equipment, health monitoring systems for social infrastructure, and medical and health applications.

## 1. Introduction

Vacuum monitoring technology for industrial equipment is becoming increasingly important. With the boom in the semiconductor market, the relative industry has become increasingly dependent on vacuum technology [1,2,3]. Since the invention of the Pirani vacuum gauge in 1906, it has been widely used in various application fields because of its low cost and easy operation [4]. The principle of the Pirani vacuum gauge is a well-known subject. In the gauge, a heating resistance wire is suspended in a gas environment and dissipates heat to gas molecules [5,6]. The vacuum degree changes the number of molecules that can increase or decrease heat dissipation. Hence, measuring heat dissipation is an indirect method of measuring a vacuum. Traditional Pirani vacuum sensors have underwent years of development and have been used in diverse fields [7,8,9]; however, they are also subject to constraints, such as high cost and a large size, and they are difficult to mass-produce. The state of the art and development of microelectromechanical system (MEMS) technologies have revolutionized various industries, especially vacuum sensor technology [10,11,12]. The MEMS-based Pirani sensor not only has a low production cost and the ability to save power consumption [13,14], it also facilitates easy integration in measurement systems [15,16]. 

Although many high-performance Pirani vacuum gauges based on MEMS techniques have been developed, they usually have a relatively complex structure and a large size [17,18]. To adapt to the requirements of more specified application fields, the MEMS Pirani sensors should be as compact as possible for large-scale arrays at a low cost. This study sought to develop a MEMS Pirani sensor that can afford in-situ measurements of uneven pressure distributions (mainly within 1–100 Pa) in narrow working spaces, while exhibiting quick responses. An ultra-compact MEMS Pirani vacuum sensor was developed in this study via a simple MEMS fabrication process. All structures of the proposed sensor were constructed using only four photomasks with the lift-off processes throughout the production, except for the back release in the final step. 

## 2. Experimental of the MEMS Pirani Sensor

### 2.1. MEMS Pirani Sensor Conception

**Figure 1:**
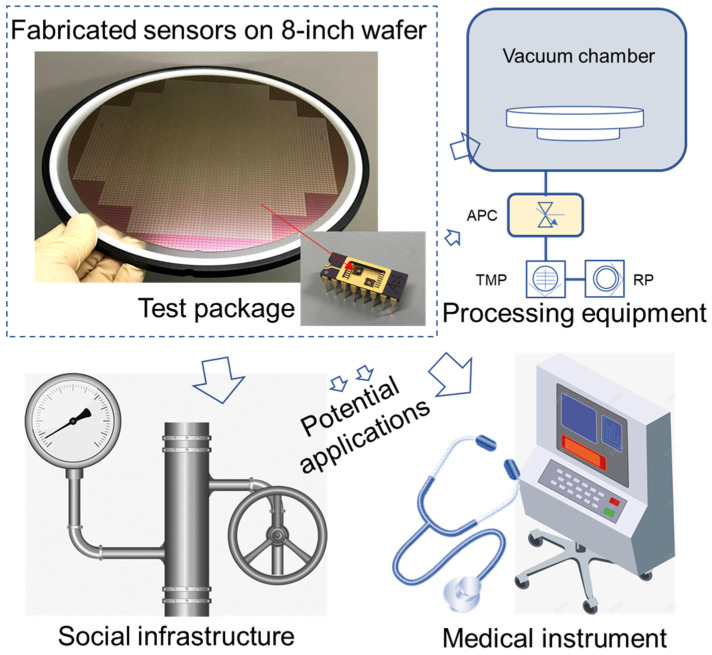
Conception and potential application of the MEMS Pirani vacuum sensor. Inserts are an optical image of the sensors fabricated on the eight-inch wafer and a package sensor for evaluation.

Figure 1 shows the conception and potential application of the proposed MEMS Pirani sensor. We designed a series of super-miniaturized MEMS Pirani vacuum sensors based on the simulation calculation results. The sensor was successfully fabricated on an 8-inch MEMS production line. We performed a comprehensive evaluation of the sensor, and the microstructures of the fabricated vacuum sensor were observed using scanning electron microscopy (SEM). After completing the test package and device signal processing circuit, the output voltages of the fabricated sensor devices with different resistor lengths under different pressures were evaluated comprehensively. We measured the internal resistance changes and temperature increase of the proposed sensor resistor caused by applying different voltage and pressure values. The developed MEMS Pirani vacuum sensor has several advantages. It is based on the MEMS process enabling it to have compact die size for saving space in the sensor terminal assembly, which will reduce costs. Moreover, the sensor can be fabricated using a simple process, which further reduces costs. According to the test results, the fabricated sensor has a large working range, from atmosphere pressure to <0.1 pascals. Thus, the sensor can be used in many applications. Owing to its cost effectiveness, the MEMS Pirani vacuum sensor can be considered for many applications, such as vacuum measurements for processing equipment, health monitoring systems for social infrastructures, and medical and health applications. 

### 2.2. Sensor Design

**Figure 2:**
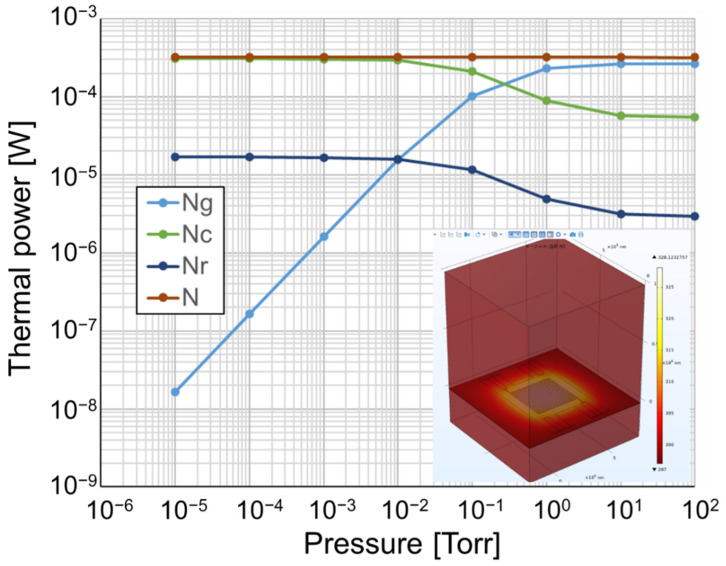
Calculation results of the thermal power of a typical sensor. The inset shows the simulation results of the temperature of a sensor with longest resistor design, indicating its temperature increase for an applied voltage.

Calculations and finite element method (COMSOL Multiphysics, COMSOL Inc. Burlington, MA, USA) were used to validate the structure segment of the Pirani vacuum sensor. Figure 2 shows the calculation results of the thermal power of a typical sensor structure. N_g_, N_c_, and N_r_ denote the sensing elements that lose heat by gas convection, solid conduction, and radiation, respectively. The calculation provides the design guidelines for obtaining the desired structure by adjusting the size and parameters of the sensor. In the simulation process, the geometrical and characteristic parameters of the sensor components and materials were set. The following structural materials were used: platinum (Pt) for the sensor resistor and extended electrode and silicon nitride (Si_3_N_4_) for thermal insulation. The key parameters of the sensor structural materials were as follows: the thickness of Pt and Si_3_N_4_ were set to 200 and 300 nm, and their thermal conductivities to 71.0 and 20.0 W/mK, respectively. The density of Pt and Si_3_N_4_ were 21,450 and 3100 kg/m^3^ and the electrical conductivities were 8.9E6 and 0 S/m, respectively. The vacuum loading conditions for different sensor designs, which had different lengths of Pt resistors from 860 to 8600 μm, were set at simulated pressures from 1000 hPa to high vacuum. 

The inset shows that the simulation results of the temperature of a typical sensor increase under an applied voltage. The core components of the vacuum sensors, which are the resistor, heat-insulating layer, and beams, are comprehensively modeled and simulated. On a flat surface, the border area is 1 × 1 mm^2^, slightly larger than the heat-insulating layer and beams. In the longitudinal direction, because the main heat exchange of the sensor occurs on the upper surface, the simulation boundaries are set as 1 mm above the front side and 0.3 mm above the back side. A sufficient boundary can approximate the actual thermal diffusion conditions around the sensor, and the simulation accuracy of the sensor can be improved accordingly. The simulation results show that the surface temperature of the sensor with the longest resistor increases to 55 °C in atmospheric conditions. 

**Figure 3:**
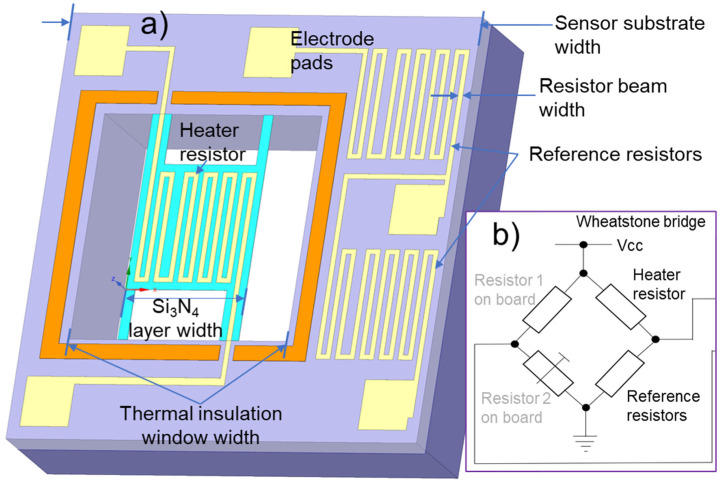
Schematic view of the Pirani vacuum MEMS sensor design with surrounding circuit. (**a**) Pirani vacuum MEMS sensor design; and (**b**) schematic view of the sensor’s surrounding circuit.

Figure 3 shows the schematic view of the Pirani vacuum MEMS sensor design with the sensor’s surrounding circuit (Figure 3b). A Wheatstone bridge was used as a detection circuit to measure the change in the resistance of the heater resistor due to temperature/vacuum changes. The heater and reference resistors are designed with the same pattern. In the Wheatstone bridge, the ratio between the heater resistor and reference resistor is 1:2 by connecting two reference resistors into one unit. The heater and reference resistors on the sensor chip and the variable resistors were packaged on the Wheatstone bridge balance adjustment and zero-point debugging circuit board. We assume that the heater resistor can generate a specific temperature difference when the vacuum value changes. In addition, the absolute temperature of the heater would not be so high as to reduce the service life. Thus, the critical structure of the proposed sensor and the temperature rise of heater resistors with different resistance values in different vacuum conditions are simulated. All the simulations and calculations aim to devise optimized design principles for realizing the sensor with an ultra-compact size. The distribution of the temperature field of the Pirani sensor was considered, and the optimized width size of the thermal insulation Si_3_N_4_ layer and the insulation window can be determined at 500 μm and 900 μm, respectively. The resistor beam width was determined to be 8 μm by considering the desired sheet resistance of the proposed resistor and the reasonable accuracy and stability of MEMS fabrication. The simulation results may exhibit a deviation error from the actual measurement results. Under the influence of various factors such as the constraints, the simulation environment deviates from experimental conditions. The two main influencing factors in this deviation are the deviation of the material parameters and dimensions of the sensor. The material parameters set in the simulation are the parameters in the COMSOL material library, ideal values that inevitably deviate from the actual values in the fabricated device. In contrast, the dimensions of the vacuum sensor in the simulation follow the design size entirely, and the fabricated sensor structure size is slightly affected by MEMS processing. Therefore, the simulation process provides a trend reference for sensor design. After the sensor fabrication trial and comprehensive evaluation, the measurement results should be used to check and improve the simulation process. Thus, the ideal model using the design guideline can be approached efficiently and accurately.

### 2.3. MEMS Fabrication Processes

**Figure 4:**
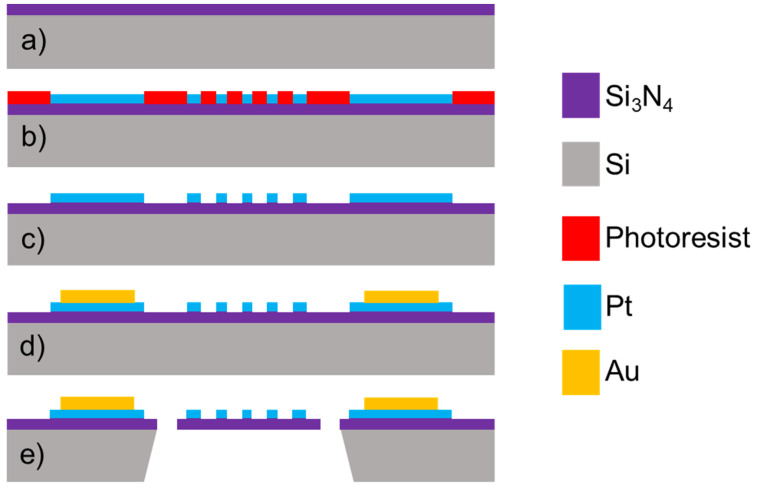
Schematic fabrication sequence of the MEMS Pirani vacuum sensor. (**a**) Si_3_N_4_ layer generation; (**b**) metallic electrode sputtering; (**c**) lift-off processes with photoresist; (**d**) Au-pads generation and patterning; and (**e**) sensor structure release.

The sensor fabrication process started with an 8-inch and 300-μm thickness Si wafer. The device fabrication was performed by the MEMS process using 4 photomasks shown in Figure 4 and described as follows:

1. After the initial wafer cleaning and passivation-layer generation, a 300-nm heat-insulating layer of Si_3_N_4_ was generated on the top surface of the Si wafer by using a vertical furnace equipment (VF-3000, Koyo Co., Ltd. Tenri, Nara, Japan);
2. The lift-off photoresist used for the Pt layer was uniformly coated on the surface and well patterned; a sputtering equipment (SME-200E, Ulvac Co., Ltd. Chigasaki, Kanagawa, Japan) was used to deposit the Pt and gold (Au) metal films on the wafer. The layers of Ta_2_O_5_/Pt with a thickness of 20 nm/200 nm were generated on the top surface of the photoresist, and the resistor and electrode pads were fabricated by a lift-off process;
3. After Au layer lift-off photoresist coating and patterning, a 500-nm-thickness Au layer was generated on the Pt electrodes for wire and package bonding;
4. The Si_3_N_4_ layer was patterned by dry etching, and Si was released by wet etching from the backside. To pattern the Si_3_N_4_ layer and remove the photoresist layer and other residuals after all the processes, dry etching was performed (E-628, Panasonic Co., Ltd. Moriguchi, Osaka, Japan). Finally, O_2_ plasma was used to remove the amorphous fluoropolymer material and other residuals.

### 2.4. Evaluation Methods

**Figure 5:**
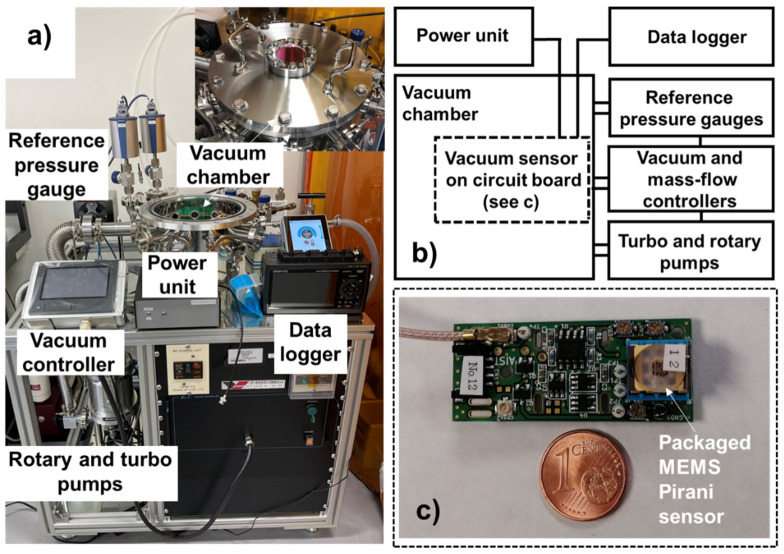
(**a**) MEMS Pirani vacuum sensor evaluation system and measurement board. (**b**) Optical photograph and configuration flow chart of the evaluation system. (**c**) Optical photograph of the packaged vacuum sensor set on the measurement board.

Figure 5 shows the evaluation system for the MEMS Pirani vacuum sensor and measurement board. Figure 5a,b show the optical photograph and flowchart, respectively, of the measurement system used to evaluate the vacuum sensor. First, an oil rotary vacuum pump (GLD-100A, Ulvac Co., Ltd. Chigasaki, Kanagawa, Japan) and a turbo molecular pump (TH350, Osaka Vacuum Industrial Co., Ltd. Osaka, Japan) were set at the front of the test system, which was turned on and operated continuously. The rotary vacuum pumps were connected to the vacuum chamber through the pipe. The vacuum degree in the vacuum chamber was controlled and adjusted using the vacuum controller (APCS-001, All Vacuum Create Co., Ltd. Hitachinaka, Ibaraki, Japan) and mass-flow controller (FCST1005LC, Fujikin, Co., Ltd. Osaka, Japan). A low-noise power supply unit was used to provide power to the sensor, and a data logger (DL, Hv gl2000, Graphtec Co., Ltd. Yokohama, Kanagawa, Japan) was used to record the results. An analog pressure full-range cathode Pirani gauge (M-361CP-SP/N25, Ulvac Co., Ltd. Chigasaki, Kanagawa, Japan) and two capacitance manometers (CDG045Dhs, Inficon Co., Ltd. Kawasaki, Kanagawa, Japan) were used to monitor the pressure in the chamber as a reference pressure gauge. Figure 5c is the optical photograph of the packaged vacuum sensor set on the measurement board. A 1 euro cent coin is used to compare with the measurement board to highlight the compact size of the proposed sensor system. A high-performance instrumentation amplifier (InAmp, AD8429, Analog Devices Co., Ltd. Wilmington, Massachusetts, USA) was used as the measurement circuit. In reasonable circuit designs, the amplifier can provide 4 signal amplification functions, namely 1-, 10-, 100-, and 1000-times, for the measurement circuit. In this study, we used the 10-times amplification function for sensor system evaluation.

## 3. Measurement Results and Discussion

### 3.1. SEM Observation

**Figure 6:**
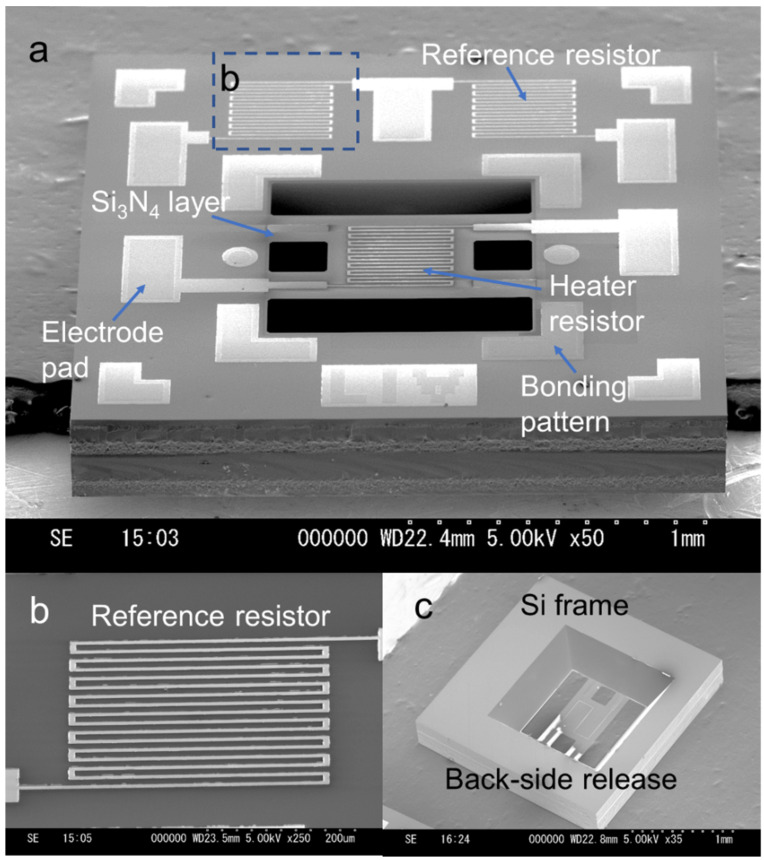
(**a**) SEM image of the proposed vacuum sensor. (**b**) The magnified SEM image of a reference heater with square-wave layout structures. (**c**) The sensor structure after the back side releasing process.

To evaluate the accuracy of the fabricated microstructure of the Pirani MEMS sensor, SEM (S-3000H, Hitachi Co., Ltd. Tokyo, Japan) was used to observe the fabricated vibration sensor. Figure 6a shows a SEM image of a typical vacuum sensor structure. As the figure shows, the vacuum sensor structure has an area of 2.2 × 2.2 mm^2^ and a thickness of 300 μm. The fabricated sensor layout realizes a final device die area of fewer than 5 mm^2^, which can significantly reduce the sensor production cost in foundries. The heater resistor is located on the 300-nm-thick Si_3_N_4_ square-shaped thermal insulation film, supported by four Si_3_N_4_ beam flexures. The Si_3_N_4_ square area has the same length and width of 500 μm, and the supporting beam flexure has a length of 200 μm and a width of 160 μm. The surrounding Si substrate cages the square-shaped film and supporting beam flexures. The typical vacuum sensor has a resistor on the square-shaped film and two reference resistors on the surrounding Si substrate to perform the differential operation that eliminates the effect of ambient temperature on the sensor, except for the barometric parameters. Figure 6b shows a magnified SEM image of the reference resistor on the chip. The 200-nm-thick micro–nano structure of the metallic resistor line was precisely partnered after the lift-off MEMS process. An appropriately partnered metallic resistor line can effectively suppress intense changes in local resistance and ensure the internal resistance consistency of different resistors. Figure 6c shows the backside structure of the sensor after the backside release process. The backside releasing process is etching through the wafer and precisely opening a thermal insulation window with dimensions of 900 μm × 900 μm at the front side. Figure 6c also shows the etched boundary of the surrounding Si substrate; the Si substrate materials were sufficiently etched and had a smooth surface. The compact-sized MEMS Pirani sensor was appropriately fabricated, and the proposed sensor can be effectively realized for in-situ pressure distribution monitoring in instrument systems, with a low manufacturing cost and high integration features. 

### 3.2. Vacuum Measurement for Different Size Sensor Resistors

**Figure 7:**
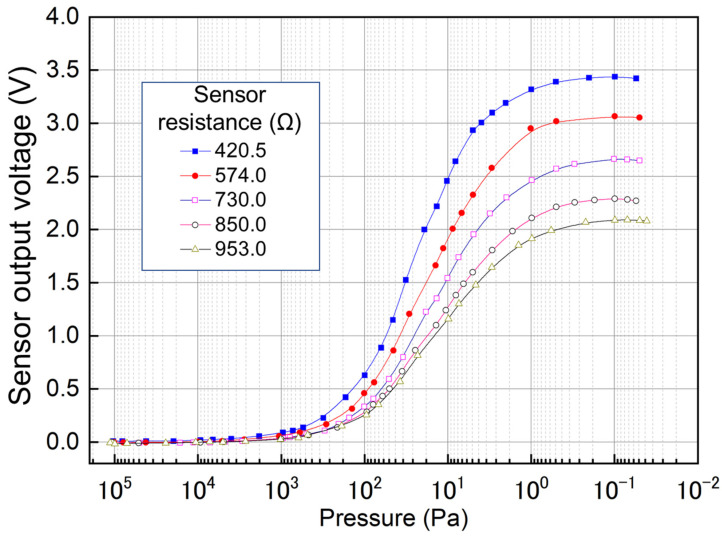
Measured output voltage of the fabricated sensor device with different resistors ranging from 420.5 to 953.0 Ω under different pressure conditions.

Figure 7 shows the measured output voltage of the fabricated sensor device for the different resistor lengths under different pressures. The voltage source was used to apply a working voltage of 3 V to the sensors. The experiment was performed at a stable room temperature of 25 °C. The performance of 5 vacuum sensors with heating resistors with resistance from 420.5 to 953.0 Ω in the air was measured. The signal test circuits set a 10× amplifier and a low-pass filter to reduce the sampling difficulty. The results demonstrate that the 5 sensors with different resistors have a reasonable response to vacuum pressures from 10,000 to 0.1 Pa. Comparing the voltage outputs of the five different sensors, the sensor with the smallest internal resistance had the highest voltage output. Because air pressure has a greater influence on temperature increase of the sensor with low internal resistance, the developed sensor system had a voltage output of 3.30 V at 1 Pa (see 420.5-Ω sensor output in Figure 7).

A careful observation of the results shows that the sensors had relatively low responsivity in the pressure range from the atmospheric pressure to 1000 Pa. The sensor’s output curve was not completely linear; this is an inherent characteristic of Pirani vacuum sensors [19]. The measured sensors had a high response domain from 100 Pa; for example, the 953.0-Ω-resistance sensor had an average responsivity of 11.9 mV/Pa in the range of 100 to 7 Pa and that of 96.0 mV/Pa in the range of 7 to 1 Pa. Compared to the standardized sensor [18], the proposed sensor has reasonable responsivity within our preferred measurement pressure range but has features such as smaller membrane and die size, and a less complex structure without a heat sink. The previous study [6] used an SOI wafer for fabrication, which has limitations on the gap thickness between the membrane and substrate, while we used an Si wafer and a backside release process. Additionally, the similar sensor [6] used Ni as the heater resistance, which naturally has higher responsivity but lesser chemical stability than Pt used in the proposed sensor. Although the proposed sensor has similar responsivity to the compared sensor, we believe that this result will be of great significance to researchers in this field. 

According to the measured results, the response domain can be divided into several ranges. The proposed Pirani sensors biased by constant voltage had different average responsivity to each pressure range, as shown in Table 1. In the high vacuum range from 0.1 to 1 Pa, the highest responsivity of the sensor with 730.0-Ω heater is up to 226.7 mV/Pa. Meanwhile, in the low vacuum from 1000–10,000 Pa, the responsivity decreased to 0.0045 mV/Pa. The proposed sensor sought to achieve the best response in a field from 1 to 100 Pa. Although the 420.5 and 574.0-Ω sensors have a slightly high average responsivity, considering that the relatively low calorific value can improve sensor system stability, the sensors with the larger resistance can realize a high-stable sensor system while ensuring a relatively high responsivity.

Experiments wherein a constant current was supplied to the sensor were also implemented. The constant-current source provided an applied current from 1 to 5 mA to the sensor at a stable room temperature of 25 °C. The sensor can reasonably respond to the atmospheric pressure up to a high vacuum. The sensor’s output is similar to the constant voltage supplying experiment, that is, among the voltage outputs of the proposed sensor with different applied current values, the sensor has the most significant output for the largest applied current. 

### 3.3. Resistance and Temperature Change of Sensor Resistor under Varying Applied Voltage and Pressure

**Figure 8:**
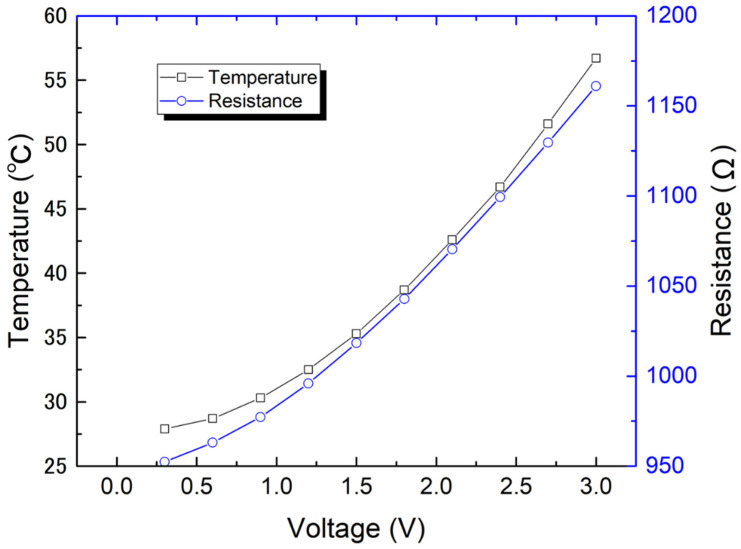
Measured surface temperature and heater resistance of the fabricated 953.0-Ω-heater sensor device for increasing the applied voltage from 0.3 to 3 V.

To directly evaluate the heating characteristics of the fabricated Pirani sensor, we also measured the resistance and temperature changes of the typical senor resistor under varying the applied voltage and pressure. A thermal imager camera (PI640, Optris Co., Ltd. Berlin, Germany) was used to measure the temperature of the proposed vacuum sensor. Figure 8 depicts the resistance and temperature change as the voltage is applied to the typical sensor resistor. The voltage source (U8002A, Keysight Technologies, Inc. Santa Rosa, CA, USA) was used to apply voltages from 0.3 to 3 V at steps of 0.3 V. The experiment was carried out in atmosphere conditions, where the ambient air pressure was 1000 hPa. Figure 8 shows that with an increase in the applied voltage, the temperature and internal resistance of the sensor resistor increase correspondingly. The rate of increase in resistance agrees with the rate of increase in temperature of the sensor resistor. The measured temperature on the surface of the sensor resistor increased from room temperature with no applied voltage to 56.7 °C for an applied voltage of 3 V. 

**Figure 9:**
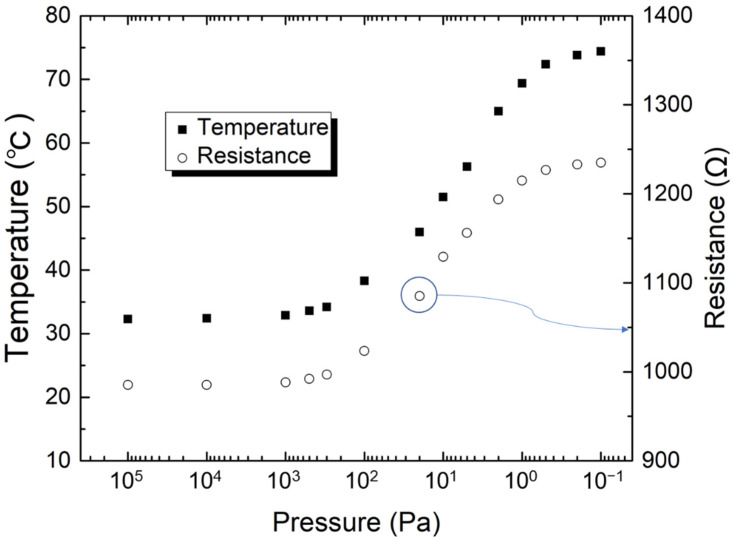
Measured surface temperature and heater resistance of the fabricated 953.0-Ω-heater sensor device for decreasing the applied pressure from 100,000 to 0.1 Pa.

Figure 9 shows the temperature and resistance changes as a function of the ambient pressure. Because the ratio between the heating and reference resistors is 1:2, the voltage source was used to provide a fixed applied voltage of 1 V. The experiment was conducted in an ambient air pressure environment from atmospheric pressure to 0.1 pa. As shown in Figure 9, the temperature and internal resistance of the typical sensor resistor increase correspondingly with a decrease in pressure. The results of membrane surface temperature experiments showed that the proposed MEMS Pirani sensor with a 953.0-Ω-heater exhibits a relatively lower surface temperature. This feature improves the safety and stability of the sensor system while reducing its power consumption. 

## 4. Conclusions

A compact MEMS Pirani vacuum sensor with a high responsivity output was successfully fabricated using a commercial eight-inch MEMS foundry process. The sensor fabrication process is a simple process that uses only four photomasks, and the sensor is fabricated with an ultra-compact size (<2.2 × 2.2 mm^2^). The fabricated Pirani vacuum sensors were evaluated comprehensively, and the results demonstrated that the MEMS Pirani vacuum sensor has a high response in the low-pressure domain from 100 Pa. The proposed sensor (with 953.0-Ω heater) system exhibited an average responsivity of 11.9 mV/Pa in the preferred range of 100 to 7 Pa and that of 96.0 mV/Pa in the range of 7 to 1 Pa. 

## Figures and Tables

**Table 1:** The average sensor responsivity derivative of the output voltage curve of Figure 7 is shown.

Heater Resistance Value	Average Responsivity of the Sensor System with 10-Times Amplification (mV/Pa)				
	0.1–1 Pa	1–7 Pa	7–100 Pa	100–1000 Pa	1000–10,000 Pa
420.5 Ω	131.1	95.5	22.0	0.630	0.0086
574.0 Ω	123.5	137.5	18.0	0.420	0.0059
730.0 Ω	226.7	113.1	15.0	0.349	0.0045
850.0 Ω	206.6	113.3	13.8	0.281	0.0035
953.0 Ω	197.1	96.0	11.9	0.248	0.0030

## Data Availability

Not applicable.
